# Wisconsin’s Health Department-University Partnership Model for Comprehensive Cancer Control

**Published:** 2009-03-15

**Authors:** Amy Ellestad Conlon, Kim Treml, Patrick Remington, Mark V. Wegner, Mary Baliker, Patrick Remington

**Affiliations:** University of Wisconsin Paul P. Carbone Comprehensive Cancer Center; University of Wisconsin School of Medicine and Public Health, Madison, Wisconsin; University of Wisconsin School of Medicine and Public Health, Madison, Wisconsin; Wisconsin Division of Public Health, Madison, Wisconsin; University of Wisconsin Paul P. Carbone Comprehensive Cancer Center, Madison, Wisconsin; University of Wisconsin Paul P. Carbone Comprehensive Cancer Center, Madison, Wisconsin

## Abstract

Cancer causes substantial morbidity and mortality every year in the United States. To address cancer prevention and control in Wisconsin, the Wisconsin Division of Public Health and the University of Wisconsin Paul P. Carbone Comprehensive Cancer Center forged a unique partnership. Using funds from the state legislature, the university, and the Centers for Disease Control and Prevention, the Wisconsin Comprehensive Cancer Control Program was created.

This health department–university partnership model has allowed both institutions to contribute their distinct strengths to projects that neither organization would have been able to complete on its own. Some challenges also have arisen during development and execution of the program, but overall, this collaborative partnership has brought diverse groups together to develop and implement evidence-based cancer control programs and policies in Wisconsin.

## Introduction

In 2003, more than 11,000 Wisconsin residents died from cancer and nearly 28,000 were diagnosed with cancer (1). Cancer was the second leading cause of death among all age groups combined, accounting for more than 20% of all deaths in Wisconsin (1). Ways to prevent and treat cancer and extend survival time and quality of life continue to be identified.

In 1998, the Centers for Disease Control and Prevention (CDC) established the National Comprehensive Cancer Control Program. The program was designed to assist states in developing a comprehensive cancer control plan that addressed their unique cancer needs. CDC defines comprehensive cancer control as "an integrated and coordinated approach to reducing cancer incidence, morbidity, and mortality through prevention, early detection, treatment, rehabilitation, and palliation" (2).

Wisconsin's Department of Health Services, Division of Public Health (DPH) shared CDC's commitment to comprehensive cancer control. However, state budget constraints were a barrier to developing a program in Wisconsin using only health department resources and staff. Therefore, the health department forged a partnership with the comprehensive cancer control center at the University of Wisconsin to develop a comprehensive cancer control program in Wisconsin.

## Historical Background

Wisconsin's cancer control history laid the foundation for the health department–university partnership and the achievements of Wisconsin's Comprehensive Cancer Control Program. Both the University of Wisconsin and the Wisconsin DPH have conducted cancer research and interventions (3). The university began conducting basic science research on cancer in 1940 at the McArdle Laboratory for Cancer Research, the first academic cancer research center of its kind in the United States. In the mid-1970s the clinical cancer center was established. In the early 1980s, Paul P. Carbone, MD, the clinical cancer center director, and Gerald Doelle, Executive Vice President of the American Cancer Society, Wisconsin Division, founded the Wisconsin Cancer Council, a consortium of statewide partners committed to cancer control, as the state's first cancer control coalition. The framework for the health department–university partnership was put in place when Dr Carbone worked with then-Governor Tommy Thompson to establish the Governor's Cancer Control Initiative in 1988. This initiative received nearly $400,000 annually from the state's general purpose revenue to support statewide cancer control initiatives (4).

On the health department side, in the mid-1970s, the Wisconsin state legislature established the Wisconsin Cancer Reporting System (5). Then, in 1987, Wisconsin was one of the first states to receive a National Cancer Institute (NCI) Data-Based Intervention Research Grant. Patrick Remington, MD, MPH, joined the health department in 1988 and assumed leadership of the grant (6). Because of a freeze in hiring state staff, the health department contracted with the university to hire 2 key staff members — an epidemiologist and a health communications expert — to work at the health department and assist in planning and implementing cancer control initiatives (7,8).

During the next 15 years, the health department and university developed a strong collaborative relationship. The health department was awarded several cancer control research grants from the NCI, such as the American Stop Smoking Intervention Study Project (9) and the Public Health Approaches to Breast and Cervical Cancer grant (10,11), with support from collaborators at the university. The university also received grants from NCI to establish the Women's Health Study, one of the largest case-control studies of breast cancer in the world, in close collaboration with colleagues at the health department (12). Throughout this time, the university continued to house the Wisconsin Cancer Council to promote statewide collaboration in cancer control.

## Partnership Development

In 2002, CDC invited cancer control leaders from each state to the first Comprehensive Cancer Control Leadership Institute. Wisconsin representatives from the health department, university cancer center, Wisconsin Cancer Council, and other organizations attended the conference and reaffirmed their commitment to cancer control. After the leadership institute, the health department wanted to apply for a comprehensive cancer control planning grant from CDC but lacked the staff and infrastructure to carry out the planning process should the grant be awarded. Although the cancer center could provide administrative support, staff, and space to house the CDC-funded cancer control program, the CDC planning grant was available only to state health agencies. The partnership between the health department and the cancer center was the basis for Wisconsin's Comprehensive Cancer Control Program. The partnership arose out of a need for infrastructure assistance on the part of the health department and a willingness to commit to public health practice, not just research, on the part of the cancer center.

## Developing the Wisconsin Comprehensive Cancer Control Plan

Although the health department and the cancer center provide the major financial and administrative infrastructure for the state's comprehensive cancer control program, its development depended on statewide partners to provide insight and expertise throughout the planning process. The Core Planning Team, made up of representatives from the health department, cancer center, Wisconsin Cancer Council, and American Cancer Society, assembled a diverse group of academic, public health, health care, and community partners to serve as the plan's steering committee. The steering committee developed the framework for the plan, including the vision, mission, priority, and crosscutting issues, and served as its decision-making body. The plan's joint leadership from the health department and cancer center attracted experts from multiple disciplines, providing diverse representation to ensure that the state's cancer control plan was truly comprehensive and carefully constructed. The Wisconsin Cancer Council's well-established network of physicians, hospitals, and community agencies served as the foundation of community partners. Because of the cancer center's involvement, academic experts were willing to participate, whereas they may have otherwise shied away from a government program. Likewise, those who may have avoided academia were drawn by the health department's involvement. This diverse group of partners has been a strength of the Wisconsin Comprehensive Cancer Control Plan from the developmental phase through its implementation phase.

While the steering committee developed the framework of the plan, the Cancer Data Advisory Group looked at Wisconsin-specific data to identify priorities and inform decision-making. The group recommended priorities for intervention that the steering committee discussed, revised, and approved (13). Once priorities were identified, workgroups were formed around the areas of prevention, screening and detection, treatment, quality of life, palliative care, and data collection and reporting. These workgroups developed strategies and action plans to address the priorities of the statewide plan. The combined work of the steering committee, the data advisory group, and workgroups became the Wisconsin Comprehensive Cancer Control Plan 2005-2010 (13).

Because the statewide program is a joint endeavor of the health department and cancer center, the plan remained an apolitical document that could be developed quickly, while at the same time being aligned with the state's strategic plan for health, *Healthiest Wisconsin 2010* (14).

## Implementing the Wisconsin Comprehensive Cancer Control Plan 

The health department–university partnership also brought additional financial resources to the effort. The statute that created the Governor's Cancer Initiative in 1987 stipulates that any organization using any part of the nearly $400,000 set aside for cancer prevention and control must provide a 1:1 match of funds (4). Together in 2005, the cancer center and the University of Wisconsin School of Medicine and Public Health provided such a match through the Wisconsin Partnership for a Healthy Wisconsin Program, an endowment established after Blue Cross and Blue Shield converted to a for-profit health insurance company. As a result, the Comprehensive Cancer Control Program had nearly $800,000 per year to dedicate to cancer control. Much of the money was given as grants to community organizations for local cancer control projects. Many of these organizations would not have been able to access money from the governor's cancer initiative fund because of the matching-funds requirement.

An example was the Healthy Lifestyles project (15). In 2006, six local nutrition and physical activity coalitions received $4,000 grants from the program. Within each coalition, these grants supported worksite wellness projects. Each local coalition worked with up to 5 local businesses to develop programs aimed at improving the activity levels and dietary habits of the adult working population. The Healthy Lifestyles project also took advantage of the culture of cooperation created through the health department–university partnership. The Comprehensive Cancer Control Program (CCCP) teamed with the health department's Nutrition and Physical Activity Program to administer the Healthy Lifestyles grants. The CCCP provided the funding and the Nutrition and Physical Activity Program provided guidance to the local coalitions through the development of a worksite wellness toolkit (16). The 2 programs continue to work together to improve Wisconsin lifestyles and reduce chronic diseases. In addition to fostering cooperation, the health department–university partnership has created a much larger network of partners that are available for cancer control.

Wisconsin's partnership model for cancer control offers practical advantages to both the health department and university. Expanded resources (time, staff, and money) are available for the state's comprehensive cancer control program and specific implementation projects. Students hired through the university perform administrative duties and event planning. Student assistance is not often readily available at government health departments. For implementation projects, the additional staff resources available through the university create possibilities that would otherwise not exist. The Wisconsin Cancer Reporting System border county pilot project is a prime example.

The state's cancer registry, part of the Wisconsin Division of Public Health, collects cancer data on Wisconsin residents and maintains a population-based database for public health surveillance and research (17). Because Wisconsin does not have a data exchange agreement with Minnesota, its western neighbor, cancer cases for Wisconsin residents diagnosed or treated in Minnesota are not reported to the cancer reporting system. This missing information compromises the completeness and quality of the registry, especially for counties bordering Minnesota. The cancer reporting system was not able to address the problem internally because of budget and time constraints, and improving the quality and completeness of cancer data collection is one of the state's cancer control program's priorities. By partnering with the cancer center at the university, the health department's cancer reporting system was able to implement data exchange agreements with specific Minnesota hospitals near the Wisconsin–Minnesota border. The 11 facilities that participated in this special data collection effort submitted 2,835 case reports; of those, 977 were reports of illness diagnosed from 1998 to 2002 (18). The overall age-adjusted cancer rates increased between 0.8% and 19% by county after the addition of the new case reports (18). The total rate increase for the state of Wisconsin was 0.8% (18). This project has reciprocal benefits for the cancer center. By being part of a nonresearch-focused program, the cancer center is able to offer outreach and advocacy experience to its population and public health students. Likewise, the completeness of cancer-related data benefits university researchers who use the center's data to write grants and papers.

Researchers from the University of Wisconsin's Paul P. Carbone Comprehensive Cancer Center (UWCCC) have been able to expand their own cancer control research through the partnership. One example of this is the Assessment of Cancer CarE and SatiSfaction (ACCESS) study, which was jointly supported and created by UWCCC and DPH. The overall purpose of this study was to assess cancer patients' experiences of their care, particularly regarding satisfaction with care. Participants in the ACCESS study were Wisconsin residents diagnosed with breast, colorectal, lung, and prostate cancer. The ACCESS study developed long-term data measurement criteria for comprehensive cancer control planning efforts to support improvements in patient cancer care in Wisconsin. The ACCESS study results are being analyzed and will serve as pilot data for future research into the determinants of quality of cancer care.

The partnership between the health department and university has advanced cancer-related policy issues in Wisconsin. Although the health department, as a government agency, must not take an official stand on legislative issues, the cancer center, as a separate academic institution, can offer an additional, credible voice. One example of how the cancer center's support has driven forward legislation is Nick's Law (19). This law allows cancer patients and their families to donate their unused, unopened medications and supplies to participating pharmacies that can redistribute them to those who are poor, uninsured, or underinsured. Together partners of the Wisconsin Comprehensive Cancer Control Program can advocate and support legislation like this that is in the best interest of cancer control.

## Challenges of the Partnership

The partnership between a government agency and a state university system provides a unique leadership opportunity for Wisconsin's cancer control planning and implementation efforts. The health department is rooted in public health practice, and the cancer center and university are rooted in research; their perspectives and expertise are often complementary. Yet cooperation between 2 institutions with distinct philosophical missions has been challenging at times. The most cumbersome challenges have been logistic and administrative. Funds must be managed carefully because of distinct and often conflicting administrative and budgetary processes. Coordinating staff, work, and other resources can be difficult because some people physically work at the health department and some at the university. Working in 2 institutions with different bureaucratic systems sometimes delays progress. However, Wisconsin's Comprehensive Cancer Control Program staff members at both the health department and the university are aware of these challenges and how to address them so they do not impede progress. Open communication between the partners has been the most effective way to balance the needs of both sides while staying focused on the goals of the statewide cancer plan.

## Conclusion

Overall, Wisconsin's health department–university partnership shows how the strengths of 2 separate institutions with different agendas and missions can be leveraged to achieve a common goal. It has resulted in process improvement and has allowed for the development of specific projects that may not otherwise have been possible. The Wisconsin Comprehensive Cancer Control Program can do more to improve the health of Wisconsin than either the health department or the university could do alone. The collaboration has not been without its difficulties, but the organizations have been able to rise above the challenges. This model has been an example of synergy between academic and government agencies. The health department and university continue to refine the process and develop new projects to take advantage of each other's unique strengths.
